# Accelerated intoxication of GABAergic synapses by botulinum neurotoxin A disinhibits stem cell-derived neuron networks prior to network silencing

**DOI:** 10.3389/fncel.2015.00159

**Published:** 2015-04-23

**Authors:** Phillip H. Beske, Stephen M. Scheeler, Michael Adler, Patrick M. McNutt

**Affiliations:** Cellular and Molecular Biology Branch, Research Division, United States Army Medical Research Institute of Chemical DefenseAberdeen Proving Ground, MD, USA

**Keywords:** botulinum neurotoxin, stem cell-derived neurons, SNAP-25, synaptic transmission, glutamatergic synapse, GABAergic synapse, botulism

## Abstract

Botulinum neurotoxins (BoNTs) are extremely potent toxins that specifically cleave SNARE proteins in peripheral synapses, preventing neurotransmitter release. Neuronal responses to BoNT intoxication are traditionally studied by quantifying SNARE protein cleavage *in vitro* or monitoring physiological paralysis *in vivo*. Consequently, the dynamic effects of intoxication on synaptic behaviors are not well-understood. We have reported that mouse embryonic stem cell-derived neurons (ESNs) are highly sensitive to BoNT based on molecular readouts of intoxication. Here we study the time-dependent changes in synapse- and network-level behaviors following addition of BoNT/A to spontaneously active networks of glutamatergic and GABAergic ESNs. Whole-cell patch-clamp recordings indicated that BoNT/A rapidly blocked synaptic neurotransmission, confirming that ESNs replicate the functional pathophysiology responsible for clinical botulism. Quantitation of spontaneous neurotransmission in pharmacologically isolated synapses revealed accelerated silencing of GABAergic synapses compared to glutamatergic synapses, which was consistent with the selective accumulation of cleaved SNAP-25 at GAD1^+^ pre-synaptic terminals at early timepoints. Different latencies of intoxication resulted in complex network responses to BoNT/A addition, involving rapid disinhibition of stochastic firing followed by network silencing. Synaptic activity was found to be highly sensitive to SNAP-25 cleavage, reflecting the functional consequences of the localized cleavage of the small subpopulation of SNAP-25 that is engaged in neurotransmitter release in the nerve terminal. Collectively these findings illustrate that use of synaptic function assays in networked neurons cultures offers a novel and highly sensitive approach for mechanistic studies of toxin:neuron interactions and synaptic responses to BoNT.

## Introduction

Botulinum neurotoxins (BoNTs) are highly lethal bacterial toxins produced by *Clostridium botulinum, C. butyricum*, and *C. barati* (Simpson, 2004). Of the seven BoNT serotypes (A–G) that are currently described, BoNT/A, /B, /E, and /F have been directly associated with human disease (Simpson, 2004). The toxin is expressed as a single 150 kDa peptide, which is proteolytically cleaved to produce a dichain composed of a 100 kDa heavy chain (HC) and a 50 kDa light chain (LC) linked by a disulfide bond (Singh, 2006). HC mediates binding to pre-synaptic receptors on peripheral motorneurons and autonomic neurons, facilitating toxin uptake via synaptic endocytosis. LC then translocates to the neuronal cytosol and specifically cleaves the soluble *N*-ethylmaleimide-sensitive factor attachment protein receptors (SNAREs) SNAP-25, VAMP1/2 or syntaxin-1a/b, which are functionally essential for synaptic exocytosis. Cleavage of any of these proteins prevents assembly of the conserved synaptic exocytosis complex, thereby blocking neurotransmitter release and causing the neuromuscular and autonomic symptoms associated with clinical botulism.

BoNT has been designated as a Tier 1 Select Agent because of the high lethality, lack of therapeutic options and ease of production and distribution. The combination of efficient neuronal targeting and pre-synaptic activity renders BoNTs the most potent toxins known, with estimated human lethal doses as low as 0.1–1 ng/kg (Simpson, 2004). Clinical presentation develops 2–36 h after exposure, depending on dose and route of exposure, and is characterized by a generalized weakness which progresses to a flaccid paralysis (Dembek and Alves, 2011). Botulism becomes life-threatening once the respiratory muscles are affected, requiring mechanical ventilation and supportive care to keep severely exposed patients alive. Depending on the subtype, paralysis may persist for months. Despite the severe physiological consequences of systemic intoxication, the local delivery of small quantities of BoNT has been clinically exploited for a wide array of indications. The exquisite selectivity and long duration of action have made clinical preparations of BoNT highly effective for a multitude of neuromuscular and autonomic disorders, as well as to treat chronic pain, promote wound healing, and improve facial aesthetics (Naumann et al., 2008).

Although passive immunization can be used to abstract neurotoxin from circulation, antitoxin is ineffective once toxin has bound to or internalized into neurons (Simpson, 2004). Despite identification of the intracellular target and proteolytic mechanisms of BoNT/A in 1993, attempts to develop treatments that reverse paralysis and restore normal synaptic transmission have been unsuccessful (Blasi et al., 1993; Schiavo et al., 1993; Larsen, 2009). A major contributing factor to this failure is the lack of a scalable, sensitive and relevant cell-based model of botulism that replicates *in vivo* host:toxin interactions and is suitable for therapeutic screening and mechanistic studies. While cultured cell lines such as neuroblastomas or adrenal chromaffin cells can be chemically induced to exhibit some neurotypic characteristics, these cell lines have not been found to produce functioning pre- and post-synaptic compartments (McNutt et al., 2011). Since the pre-synaptic compartment is the physiological target of BoNT, it is therefore unlikely that the full range of toxin:neuron interactions are replicated in the absence of functioning synapses. Studies involving these cell models have reported poor sensitivities to BoNTs and results have been inconsistent with *in vivo* studies and primary neuron cultures (Eubanks et al., 2007; Larsen, 2009; Hubbard et al., 2012).

Stem cell-derived neurons have recently been proposed as a next-generation platform for neurotoxin research that unifies the flexibility of cultured cell lines with the relevance of primary neurons (McNutt et al., 2011; Whitemarsh et al., 2012). Mouse embryonic stem cell-derived neurons (ESNs) replicate many unique *in vivo* characteristics of BoNT intoxication, including serotype-specific persistences, activity-enhanced onset rates and differential serotype potencies (McNutt et al., 2011; Hubbard et al., 2012). By 18 days after plating (DAP 18), ESNs exhibit spontaneously active glutamatergic synapses and action potential (AP) firing and intoxication with BoNT/A results in SNAP-25 cleavage and blockade of K^+^-evoked glutamate release (McNutt et al., 2011; Gut et al., 2013). Observations of spontaneous synaptic activity in ESNs led us to hypothesize that intoxication with BoNT/A would eliminate synaptic transmission, thereby rendering synaptic function assays as a novel method to functionally evaluate synapse- and network-level responses to intoxication. Here we test this hypothesis by using whole-cell patch-clamp recordings to evaluate longitudinal changes in synaptic transmission following addition of BoNT/A to DAP 24^+^ ESNs, and find that synaptic function assays are significantly more sensitive indicators of intoxication than are molecular readouts of SNAP-25 cleavage. The resolution of this approach was further increased by determination of time-to-50% inhibition (T50_i_) values for isolated glutamatergic and γ-aminobutyric acid (GABA)ergic synapses. The consequences of differential synapse-specific T50_i_ values to network-level behaviors were then studied using long-term current-clamp recordings. This is the first high-resolution characterization of the effects of BoNT intoxication on synaptic activity and emergent responses in neural networks. The establishment of a functionally relevant model of synaptic paralysis at physiologically relevant concentrations of BoNT is expected to accelerate mechanistic and therapeutic studies of botulism as well as of a larger array of neurotropic toxins that modulate synaptic activity and network behaviors.

## Materials and methods

### Reagents and cell culture

Unless otherwise specified, reagents for electrophysiology were obtained from Sigma-Aldrich (St. Louis, MO). Cell culture supplies for embryonic stem cell maintenance, ESN differentiation and ESN culture were obtained from Invitrogen (Carlsbad, CA). R1 murine embryonic stem cells (ATCC, Manassas, VA) were maintained, differentiated into ESNs and plated as previously described (McNutt et al., 2011; Hubbard et al., 2012; Gut et al., 2013). Experiments were conducted on ESNs between DAP 24 and 34. Data were collected from ESNs produced among 14 independent differentiations over 9 months, with no apparent change in functional responses to intoxication. With the exception of superfusion studies, BoNT/A1 (BoNT/A; specific activity = 2.5 × 10^8^ U/mg; Metabiologics, Madison, WI) was diluted to 2000 pM in appropriate buffers and added 1:100 to DAP 24^+^ ESNs for indicated durations. For binned measurements of longitudinal changes in mPSC, mEPSC, and mIPSC frequencies, each dish was incubated in ESN medium plus toxin or vehicle control until the start of the appropriate bin.

### Electrophysiology

DAP 24^+^ neuron cultures were visualized on an Olympus IX51 microscope (Shinjuku, Tokyo, Japan) equipped with a 40× lens with differential interference contrast optics. 5–10 MΩ patch pipettes were pulled from borosilicate glass (Sutter Instruments, Novato, CA), backfilled with intracellular recording buffer and dipped in Sigmacote® (Sigma) prior to use. Electrophysiology data were acquired at 20–22°C with an EPC10 (Heka, Lambrecht/Pfalz, Germany) and Heka Patchmaster 2.53 software. Current and voltage measurements were filtered online at 2.9 kHz and digitized at 10 kHz. Data analysis and graphing were performed in Heka Fitmaster 2.53, Igor Pro v6 (Wavemetrics, Portland, OR) and Prism v6.1 (Graphpad Software, La Jolla, CA).

ESNs were bathed in extracellular recording buffer (ERB) containing (in mM): 140 NaCl, 3.5 KCl, 1.25 NaH_2_PO_4_, 2 CaCl_2_, 1 MgCl_2_, 10 Glucose, 10 HEPES (pH 7.3; 315 ± 10 mOsm/Kg). Potassium-based intracellular recording buffer (K-IRB) was used to record miniature post-synaptic currents (mPSCs), miniature excitatory post-synaptic currents (mEPSCs) and cell firing activity, containing (mM): 140 K-gluconate, 5 NaCl, 2 Mg-ATP, 0.5 Li-GTP, 0.1 CaCl_2_, 1 MgCl_2_, 1 EGTA, and 10 HEPES (pH 7.3; 315 ± 10 mOsm/Kg). Cesium-based IRB (Cs-IRB) was used to record mIPSCs, containing (in mM): 140 CsCl, 5 NaCl, 2 Mg-ATP, 0.5 Li-GTP, 0.1 CaCl_2_, 1 MgCl_2_, 1 EGTA, and 10 HEPES (pH 7.3; 315 ± 10 mOsm/Kg). All traces are compensated for junction potentials of 15.6 mV (K-IRB) or 4.6 mV (Cs-IRB).

Resting membrane potential (RMP) was determined using the zero-current-clamp method immediately after establishment of whole-cell configuration. AP firing was evoked by current injection (1 s; −20 to 120 pA in 10 pA steps). Input resistance was estimated by calculating the slope of the I-V relationship determined from hyperpolarizing currents steps (1 s; −60 to 0 pA in 10 pA steps). Time constants were determined by averaging the single exponential decay rate (AxoGraph X, Berkeley, CA) of the −40 and −30 pA current steps (1 s). Rheobase was determined by the amount of current necessary to elicit the first AP using a linear ramp of depolarizing current injection (10 s; 0–200 pA). Single AP properties (threshold, AP amplitude, afterhyperpolarization amplitude) were measured on the first AP elicited in rheobase recordings using the FitMaster linear measurement tool.

To record mPSCs, ERB was supplemented with tetrodotoxin (TTX; 5 μM) to eliminate APs. To record mEPSCs, ERB was supplemented with TTX (5 μM), bicuculline (10 μM), and CGP55845 (1 μM) to eliminate APs, GABAR_A_ and GABAR_B_ currents, respectively (Curtis et al., 1970; Deisz, 1999). To record mIPSCs, TTX (5 μM), 6-cyano-7-nitroquinoxaline-2,3-dione (CNQX; 10 μM), and (2R)-amino-5-phosphonopentanoate (APV; 50 μM) was added to ERB to block APs and eliminate AMPAR and NMDAR currents (Davies and Watkins, 1982; Long et al., 1990). Miniature currents were recorded over a 3 min period in voltage-clamp mode at holding potentials of −85.6 mV (K-IRB) or −74.6 mV (Cs-IRB). Holding currents were between −25 and 0 pA. Spontaneous synaptic events were detected and quantified in MiniAnalysis using default AMPAR or GABAR_A_ detection parameters. The number of miniature currents during each recording was quantified and converted to Hz. Unless otherwise indicated, frequencies were normalized to control data collected from age- and lot-matched neurons. Kinetic parameters of miniature events were determined in MiniAnalysis from 42 neurons for mEPSCs (averaged from over 2400 events per neuron) and from 23 neurons for mIPSCs (averaged from over 400 events per neuron). Peak-scaled traces were averaged from over 100 mEPSC or mIPSC events.

For agonist-induced ionotropic glutamate receptor (iGluR) and GABA receptor (GABAR) currents, at least four neurons per treatment were superfused with agonists or inhibitors diluted into ERB using a three-barrel Fast Step system (Warner Instruments, Hamden, CT) and recorded in voltage-clamp mode. AMPA (100 μM) currents were measured in the presence of TTX (5 μM), APV (50 μM), and bicuculline (10 μM). NMDA (50 μM) currents were recorded with TTX (5 μM), CNQX (10 μM), and bicuculline (10 μM) in ERB containing 0 or 1 mM Mg^2+^, as described. Muscimol (100 μM)currents were recorded in the presence of TTX (5 μM), APV (50 μM), and CNQX (10 μM). Baclofen (10 μM) currents were recorded with TTX (5 μM), CNQX (10 μM), APV (50 μM), and bicuculline (10 μM). iGluR and GABAR_B_ currents were recorded at a holding potential of −85.6 mV (K-IRB). GABAR_A_ currents were recorded at a holding potential of −74.6 mV (Cs-IRB).

For continuous current-clamp recordings, additional drugs were not added to the ERB unless indicated. To measure spontaneous action potentials (AP) and excitatory post-synaptic potentials (EPSP), 3–4 min current-clamp recordings were conducted at RMP. Spontaneous APs and EPSPs were detected using MiniAnalysis v6 (Synaptosoft Inc., Fort Lee, NJ) with default detection settings and thresholds of 25 mV for AP detection and 5 mV for EPSP detection. For long-duration current-clamp recordings, APs were recorded from patched neurons in 6-cm dishes initially containing 2 mL ERB. After the first 10 min, 6 mL of ERB containing vehicle, toxoid, heat-denatured BoNT/A or active BoNT/A was superfused at 1.5–2 mL/min via gravity-fed plastic tubing to a final concentration of 20 pM (BoNT/A and heat-denatured BoNT/A) or 200 pM (BoNT/A toxoid). For burst characterization, APs were detected using the parameters described above and then bursts were identified as 3 or more APs with interspike intervals of less than 500 ms. All data were normalized to the 5 min bin immediately preceding superfusion.

For all electrophysiology experiments, recordings were accepted if the following criteria were met: RMP were between −68 and −80 mV; series resistances were less than 50 MΩ; and resistances did not vary by more than 20% of starting value throughout the recording.

### Immunoassays

Protein lysates were generated from ESN cultures by scraping in ice-cold RIPA buffer, quantified by BCA (Pierce), separated by SDS-PAGE and transferred to PVDF. Blots were stained and imaged as previously described (Hubbard et al., 2012). Primary antibodies used for Western blotting included SNAP-25 (Covance, Princeton, NJ) and syntaxin-1 (Synaptic Systems, Gottingen, Germany). For immunocytochemistry, ESNs on 18-mm coverslips were fixed in 4% paraformaldehyde, permeabilized with 1% saponin in TBS (TBS-S), treated with primary and secondary antibodies diluted in TBS-S and imaged by confocal microscopy as previously described (Mesngon and McNutt, 2011; Hubbard et al., 2012; Gut et al., 2013). Images were collected on a Zeiss LSM-700 confocal microscope with a 40× objective. Primary antibodies against Tau (Synaptic Systems), MAP2 (Synaptic Systems), wild-type SNAP-25 (Covance), the cleaved form of SNAP-25 (cSNAP-25; Research and Diagnostic Antibodies, Las Vegas, NV), synapsin 1A/B (SYN1; Synaptic Systems), vesicular glutamate transporter 2 (VGLUT2; Synaptic Systems), and glutamate decarboxylase 1 (GAD1; Synaptic Systems) were used for immunocytochemistry studies at manufacturer recommended dilutions. For quantification of synapse subtypes, 8 coverslips of DAP 24^+^ ESNs were triple stained for expression of SYN1, VGLUT2 and GAD1 and fluorescent co-localization of these three markers was evaluated on 250–500 synapses per coverslip (≥2000 synapses in total). Quantitation of cSNAP-25 co-localization with VGLUT2 and GAD1 was performed in four coverslips per timepoint, with at least 500 VGLUT2 and 500 GAD1 synapses counted per coverslip and scored as cSNAP-25^+^ or cSNAP-25^−^. All immunocytochemistry quantitation studies were conducted using three investigators blinded to the experimental setup.

### Statistical analysis

Spontaneous activity and mEPSC/mIPSC frequencies were calculated by determining the mean frequency (in Hz) of events measured during continuous recordings among ≥8 neurons in each condition. Rates measured in BoNT/A-treated neurons were normalized to mean rates observed in age- and lot-matched vehicle-treated neurons and presented as percentages of control rates. Normalization of inter-burst intervals and the number of APs/burst was conducted in a similar fashion, with the 5 min of recording immediately preceding superfusion used as baseline for normalization. In most cases, statistical significance among means was determined using One-Way ANOVA testing and *P*-values were calculated against controls with the Dunnett's *post-hoc* test. For long-duration current-clamp recordings, statistical significance among normalized grouped means was determined using Two-Way ANOVA and *P*-values were calculated with Bonferroni's *post-hoc* test. In binary comparisons Student's *t*-test was used to determine significance between means. For comparison of the distribution of glutamatergic vs. GABAergic synapses, Shapiro-Wilk normality test was first performed to confirm that data were normally distributed, followed by a one-sample *t*-test comparing each population against a theoretical mean of 50%. Unless otherwise stated, quantitative data are presented as mean ± the standard error of the mean, with the following levels of statistical significance: ^*^ indicates a *P* < 0.05; ^**^ indicates a *P* < 0.01; ^***^ indicates a *P* < 0.001.

## Results

### ESN cultures produce excitatory and inhibitory synapses with emergent network responses

**Figure 1:**
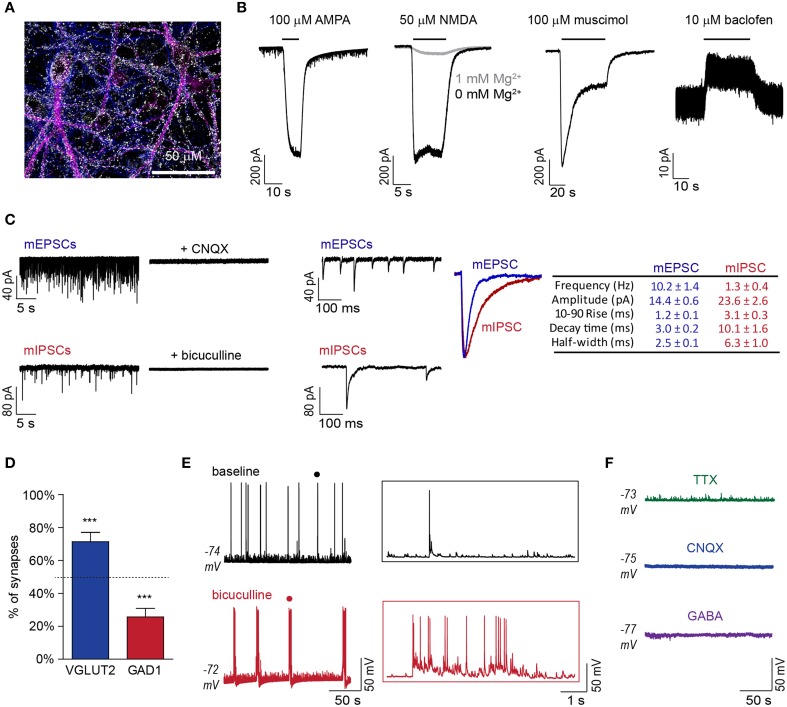
**DAP 24^+^ ESNs produce functional glutamatergic and GABAergic synapses with emergent network behavior. (A)** Representative ICC demonstrating expression of the pre-synaptic marker SYN1 (white) at regions of axodendritic interface at DAP 24 (axonal marker Tau, blue; somatodendritic marker MAP2, magenta). **(B)** Representative voltage-clamp recordings showing receptor-mediated currents following superfusion with AMPA (100 μM), NMDA (50 μM) with or without Mg^2+^ in the extracellular buffer, muscimol (100 μM) or baclofen (10 μM; *n* ≥ 4 each). **(C)** Detection of mESPCs in the presence of TTX (5 μM), bicuculline (10 μM), and CGP55845 (1 μM) and mIPSCs in the presence of TTX (5 μM), CNQX (10 μM), and APV (50 μM) on compressed (left) and expanded (middle) time-scales. CNQX (10 μM) or bicuculline (10 μM) eliminated mEPSCs or mIPSCs, respectively. Peak-scaled overlay and table (right) summarize respective kinetic parameters (*n* = 11 each). **(D)** Distribution of excitatory and inhibitory synaptic markers that co-localized with SYN1 puncta (mean ± SD; *n* = 8). ^***^ indicates *P* < 0.001 vs. theoretical mean of 50% (dashed line). **(E)** Representative current-clamp recordings of APs and EPSPs under baseline conditions and following bicuculline (10 μM) addition (left). Expanded time-scale insets (right) are from region marked by • in original recordings. **(F)** Representative current-clamp recordings following addition of TTX (5 μM), CNQX (10 μM) or GABA (100 μM; *n* ≥ 4 each).

DAP 24 ESNs exhibit polarized compartments and protein localization consistent with synapse formation, including the presence of synaptic puncta at axodendritic interfaces (Figure 1A), and express transcripts specific for glutamatergic and GABAergic lineages (Hubbard et al., 2012). However, ESNs have not been functionally characterized for expression of post-synaptic receptors, onset of synaptic activity or emergence of network behaviors. To evaluate the functional expression of glutamate and GABA receptors, agonist-induced currents were evaluated by whole-cell patch-clamp recordings in voltage-clamp mode. Bath addition of α-amino-3-hydroxy-5-methyl-4-isoxazolepropionic acid (AMPA) resulted in large inward currents, while the addition of *N*-methyl-D-aspartate (NMDA) resulted in large currents only when Mg^2+^ was absent from the extracellular solution (Figure 1B). Perfusion with the ionotropic GABA_A_ receptor (GABAR_A_)-agonist muscimol resulted in a large desensitizing Cl^−^ current, while the metabotropic GABA_B_ receptor (GABAR_B_)- agonist baclofen produced a sustained hyperpolarizing current (Figure 1B).

Miniature excitatory post-synaptic currents (mEPSCs) and miniature inhibitory post-synaptic currents (mIPSCs) were observed in DAP 24^+^ ESNs with mean frequencies of 10.2 ± 1.4 and 1.3 ± 0.4 Hz, respectively (Figure 1C). Miniature events exhibited kinetic values consistent with glutamatergic and GABAergic post-synaptic currents and were eliminated by addition of CNQX or bicuculline, respectively (Figure 1C). Characterization of synapse subtypes was simultaneously conducted by immunocytochemistry (ICC) co-localization of three markers: the universal pre-synaptic marker synapsin I (SYN1), the glutamatergic pre-synaptic marker VGLUT2 and the GABAergic pre-synaptic marker GAD1. Co-localization studies confirmed that 86.1% of SYN1 puncta could be unambiguously associated with VGLUT2 or GAD1. Of these, 76.3 ± 4.4% were co-localized with VGLUT2 while 23.7 ± 4.5% were co-localized with GAD1 (Figure 1D, S1). Frequent spontaneous excitatory post-synaptic potentials (sEPSPs), APs and short bursts typically comprised of 2–10 APs were evident in current-clamp mode, suggestive of synaptically driven network behavior. Network behavior was further evaluated by superfusion of the GABAR_A_ antagonist bicuculline, which converted stochastic AP firing to prolonged epileptiform discharges (Figure 1E). Addition of TTX eliminated spontaneous APs, while addition of CNQX or GABA eliminated all APs and EPSPs (Figure 1F). The lack of spontaneous APs and sEPSPs in the presence of CNQX suggests that APs result exclusively from excitatory synaptic activity. Collectively, these data demonstrate the functional development of glutamatergic and GABAergic synapses and the emergence of emergent network behaviors with characteristics of an excitatory/inhibitory (E/I) balance.

### BoNT/A intoxication eliminates network activity

**Figure 2:**
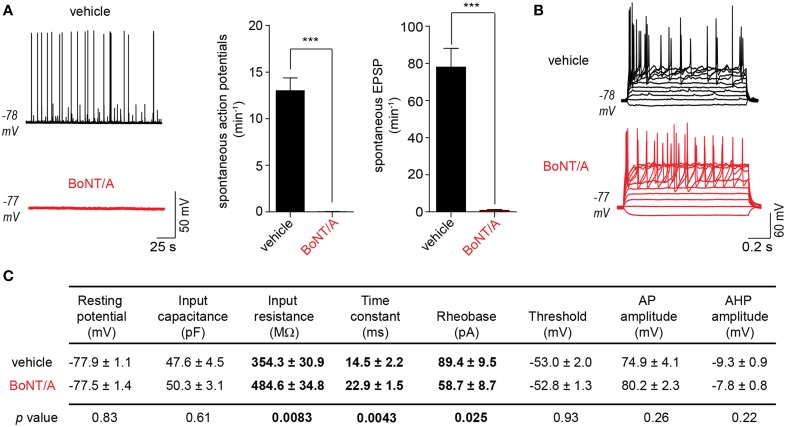
**Intoxication of DAP 24^+^ ESNs with BoNT/A eliminates synaptically driven EPSPs and AP firing. (A)** Representative current-clamp recordings of ESNs (left) 20 h after application of vehicle (top) or 20 pM BoNT/A (bottom). BoNT/A dramatically reduces the frequency of spontaneous APs and sub-threshold EPSPs compared to vehicle-treated controls (*n* ≥ 18 each). ^***^ indicates *P* < 0.001. **(B)** Both vehicle-treated and BoNT/A-treated ESNs fire repeated APs in response to depolarizing current injection (−20 to 120 pA, 10 pA steps; *n* = 18 each). **(C)** Summary of passive and active membrane properties of vehicle- and BoNT/A-treated ESNs (*n* = 18 each). *P*-values are indicated for each pairwise comparison, with significant differences in bold.

Cleavage of the essential SNARE protein SNAP-25 by BoNT/A prevents neurotransmitter release from the pre-synaptic compartment in peripheral neurons. In theory, BoNT/A-intoxication of a neuronal network would therefore reduce or eliminate synaptically driven network responses. To evaluate the effects of BoNT/A on network behaviors, we quantified spontaneous rates of EPSPs and APs at 20 h after the addition of 20 pM BoNT/A. This combination of time and dose has been found to cause the cleavage of ~95% of total cellular SNAP-25 in ESNs (McNutt et al., 2011). In current-clamp recordings, intoxication with BoNT/A eliminated over 99.1% of EPSPs and 99.8% of APs compared to vehicle-treated controls (Figure 2A). To confirm that the reduction of APs was caused by the loss of synaptic drive rather than decreased intrinsic excitability, passive, and active neuronal properties were compared between vehicle- and BoNT/A-treated neurons (Figures 2B,C, S2). BoNT/A did not elicit changes in RMP or capacitance and had no effect on the ability of intoxicated neurons to fire APs in response to current injection. However, intoxicated neurons exhibited increased excitability, as demonstrated by increased membrane resistance and reduced rheobase. Since these differences increase the likelihood that neurons will fire APs in response to post-synaptic events, they cannot be responsible for elimination of spontaneous EPSPs and APs in BoNT/A-treated ESNs. Therefore, these data suggest that BoNT/A-mediated loss of spontaneous activity is specifically attributable to impaired synaptic neurotransmission.

### Temporal characterization of the molecular and functional responses to BoNT/A intoxication in networked neuron cultures

**Figure 3:**
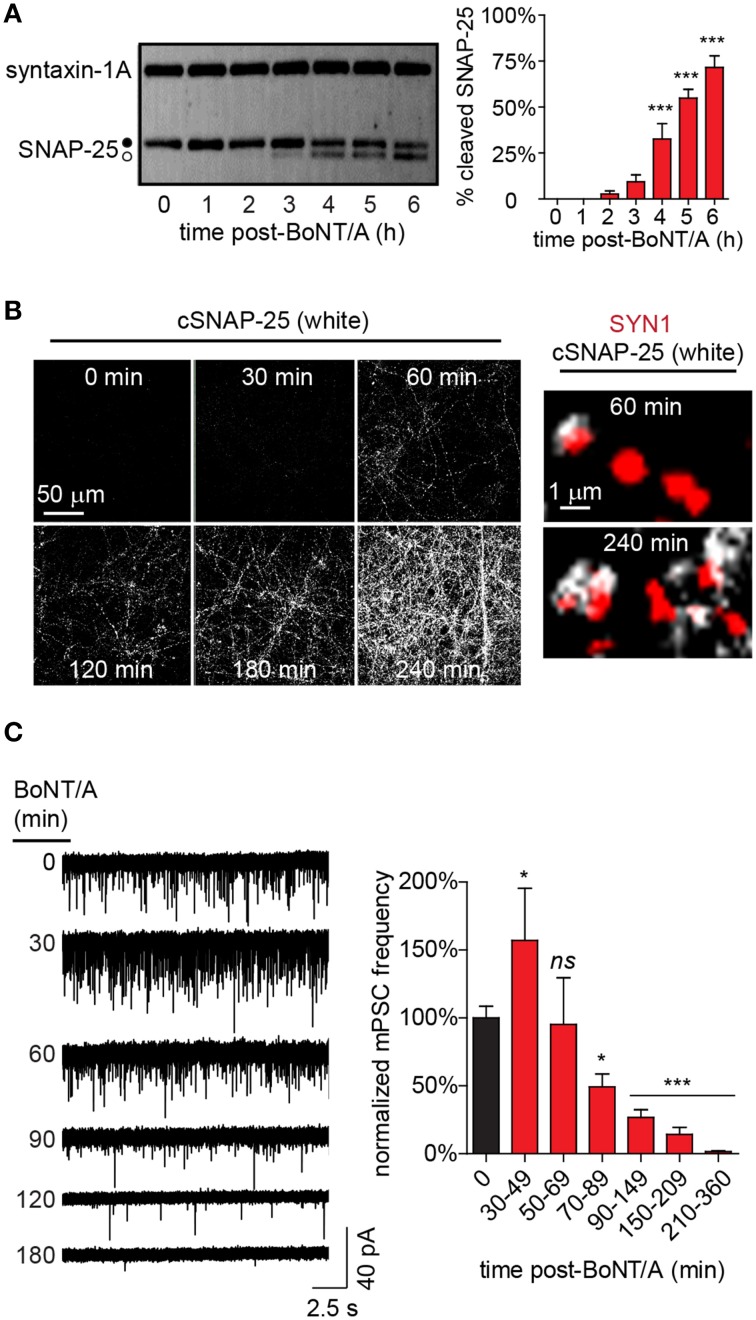
**Time-dependent changes in cSNAP-25 production and mPSC frequency following BoNT/A addition. (A)** Representative western blot (left) showing conversion of intact SNAP-25 (•) to cSNAP-25 (°) as a function-of-time after addition of 20 pM BoNT/A. The percentages of total SNAP-25 that is converted to cSNAP-25 at each time point is presented at right (*n* = 4 per timepoint). **(B)** Representative ICC of cSNAP-25 from 0 to 240 min using an antibody specific for the cleaved product (left; *n* = 3 for each timepoint). Higher magnification images (right) show co-localization of cSNAP-25 (white) with the pre-synaptic label SYN1 (red). **(C)** Representative voltage-clamp recordings performed in the presence of TTX (5 μM) at indicated time points following treatment with 20 pM BoNT/A (left). Summary of normalized mean mPSC frequencies (right; *n* = 24 controls; *n* ≥ 8 at each timepoint after BoNT/A addition). ^*^ indicates a *P* < 0.05; ^***^ indicates a *P* < 0.001; *ns* signifies not significant. Comparisons for significance are made against 0 min mean.

The above data demonstrate that intoxication caused near-complete silencing of network activity by 20 h after addition of BoNT/A. To narrow the temporal window during which dynamic changes in synapse- and network-level behaviors could be characterized via higher-resolution methods, the longitudinal production of cleaved SNAP-25 (cSNAP-25) was used as a molecular readout of intoxication from 0 to 6 h after bath addition of 20 pM BoNT/A (Figure 3A). In gel mobility-shift assays, conversion of 2.7 ± 1.6% of total SNAP-25 to the BoNT/A-cleaved product was first detectable by 2 h, followed by a progressive increase to 71.5 ± 8.2% at 6 h. To determine whether cSNAP-25 was specifically associated with nerve terminals, co-localization of cSNAP-25 with SYN1 was evaluated by ICC from 0 to 4 h after BoNT/A addition (Figure 3B). cSNAP-25 progressively accumulated at SYN1^+^ puncta from 1 to 4 h, suggesting that LC was active in pre-synaptic compartments within an hour after BoNT/A addition.

Changes in the frequency of neurotransmission are typically attributable to altered pre-synaptic release probability. To test the hypothesis that impaired pre-synaptic release of neurotransmitter was responsible for the near-complete loss of spontaneous APs and EPSPs following BoNT/A addition, we developed the measured inhibition of synaptic transmission (MIST) assay. MIST is based on comparing the frequencies of miniature post-synaptic currents (mPSCs) between intoxicated neurons and vehicle-treated controls, providing a quantitative measurement of the functional effects of intoxication on synaptic release probabilities. Based on the rapid appearance of cSNAP-25 in nerve terminals, rates of synaptic transmission were measured from 30 to 360 min after bath addition of BoNT/A (Figure 3C). Surprisingly, BoNT/A addition caused a biphasic response in mPSCs, with an initial increase in frequency between 30 and 49 min (157.1 ± 38.3%) compared to controls, followed by a steady decline to near-complete silencing of synaptic activity to 1.6 ± 0.5% of control activity by 360 min. Since the only known function of LC/A is to target and cleave SNAP-25, functionally preventing neurotransmitter release, the finding that BoNT/A intoxication caused a transient increase in mPSCs at 30–49 min was unexpected.

### Biphasic responses to intoxication reflect the preferential intoxication of GABAergic synapses at early timepoints

In the above studies, monosynaptic currents were measured at the post-synaptic soma in a complex background containing both excitatory and inhibitory synaptic events. In these types of networks, production of excitatory events can be obscured by the effects of concurrent GABAergic signaling at pre-synaptic and post-synaptic compartments, resulting in a lower apparent rate of synaptic activity (Wang et al., 2003; Chalifoux and Carter, 2011). This led us to hypothesize that the transient increase in mPSCs at 30–49 min after BoNT/A addition could be caused by the temporally selective intoxication of GABAergic synapses.

**Figure 4:**
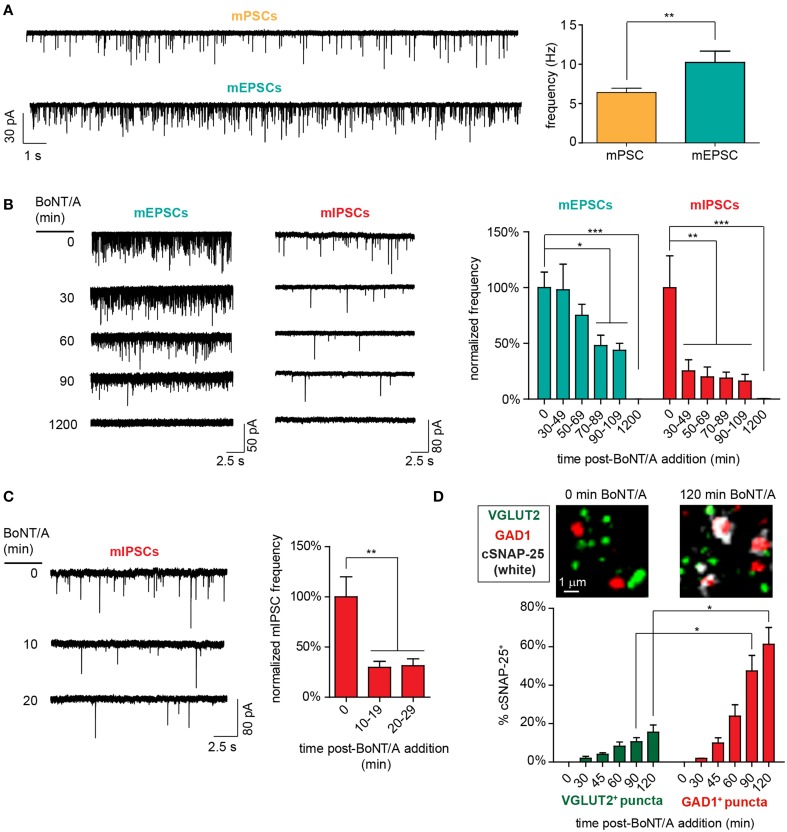
**Functional and molecular evidence of accelerated impairment of GABAergic synapses following addition of 20 pM BoNT/A. (A)** Representative voltage-clamp recordings (left) and mean event frequencies (right) demonstrating that antagonism of GABAergic signaling with bicuculline (10 μM) and CGP55845 (1 μM) increases the detection of excitatory events (*n* = 10 each). **(B)** Representative voltage-clamp recordings (left) and normalized mean frequencies (right) of mEPSC and mIPSC frequencies from 30 to 110 min after BoNT/A addition (*n* ≥ 9 per timepoint). **(C)** Representative voltage-clamp recordings (left) and normalized mean mIPSC frequencies (right) from 10 to 29 min after BoNT/A addition (*n* ≥ 10 per timepoint). **(D)** Representative ICC images (top) and percentages of GAD1^+^ and VGLUT2^+^ puncta (bottom) that are co-localized with cSNAP-25 from 0 to 120 min after BoNT/A addition (*n* = 4 per timepoint). ^*^ indicates a *P* < 0.05; ^**^ indicates a *P* < 0.01; ^***^ indicates a *P* < 0.001.

To test this hypothesis, we first determined whether GABAergic signaling influenced detection of spontaneous synaptic activity by directly comparing mPSC rates to pharmacologically isolated mEPSC rates. The addition of GABAR_A_ and GABAR_B_ antagonists increased detection of excitatory events by 160% (6.4 ± 0.6 Hz vs. 10.2 ± 1.4 Hz; Figure 4A), confirming that GABAergic activity can depress the detection of excitatory synaptic events. Intriguingly, mEPSC rates were identical to mPSC rates measured at 30–49 min after BoNT/A addition (10.2 ± 1.4 Hz vs. 10.1 ± 2.4 Hz; *p* = 0.95), suggesting that the transient increase in mPSCs at 30 min might reflect selective intoxication of GABAergic synapses. To directly determine whether GABAergic synapses were silenced more quickly than glutamatergic synapses, we measured longitudinal changes in mIPSC vs. mEPSC rates from 30 to 210 min after addition of BoNT/A (Figure 4B). While average mIPSC frequencies were reduced by 74.7 ± 10.1% as soon as 30 min after BoNT/A addition, reduction in mEPSC frequencies was not observed until 70 min after intoxication, when rates were decremented by 51.1 ± 9.3%.

To more precisely characterize the time course of GABAergic intoxication, mIPSCs were quantified in 10-min bins from 10 to 30 min after addition of 20 pM BoNT/A. Since BoNT/A internalization is mediated by activity-dependent synaptic endocytosis (Simpson, 2004), it was likely that addition of BoNT/A in the presence of TTX, which eliminates action potential-dependent synaptic activity, would significantly alter the kinetics of toxin uptake. Therefore, the 0–10 min bin was not directly evaluated using MIST. mIPSC frequency were reduced by 70.2 ± 5.9% as soon as 10–19 min after toxin addition (Figure 4C). Accelerated GABAergic intoxication was corroborated by co-localization ICC demonstrating the increased association of cSNAP-25 with GAD1 puncta vs. VGLUT2 puncta (Figure 4D). Based on these findings, the significantly shorter latency of intoxication of GABAergic synapses (T50_i_ < 10 min) compared to glutamatergic synapses (T50_i_ ≈ 70 min) offers a plausible mechanistic explanation for the transient increase in mPSC rates observed at 30–49 min after BoNT/A addition.

### Preferential BoNT/A intoxication of GABAergic synapses rapidly disinhibits neuronal networks

**Figure 5:**
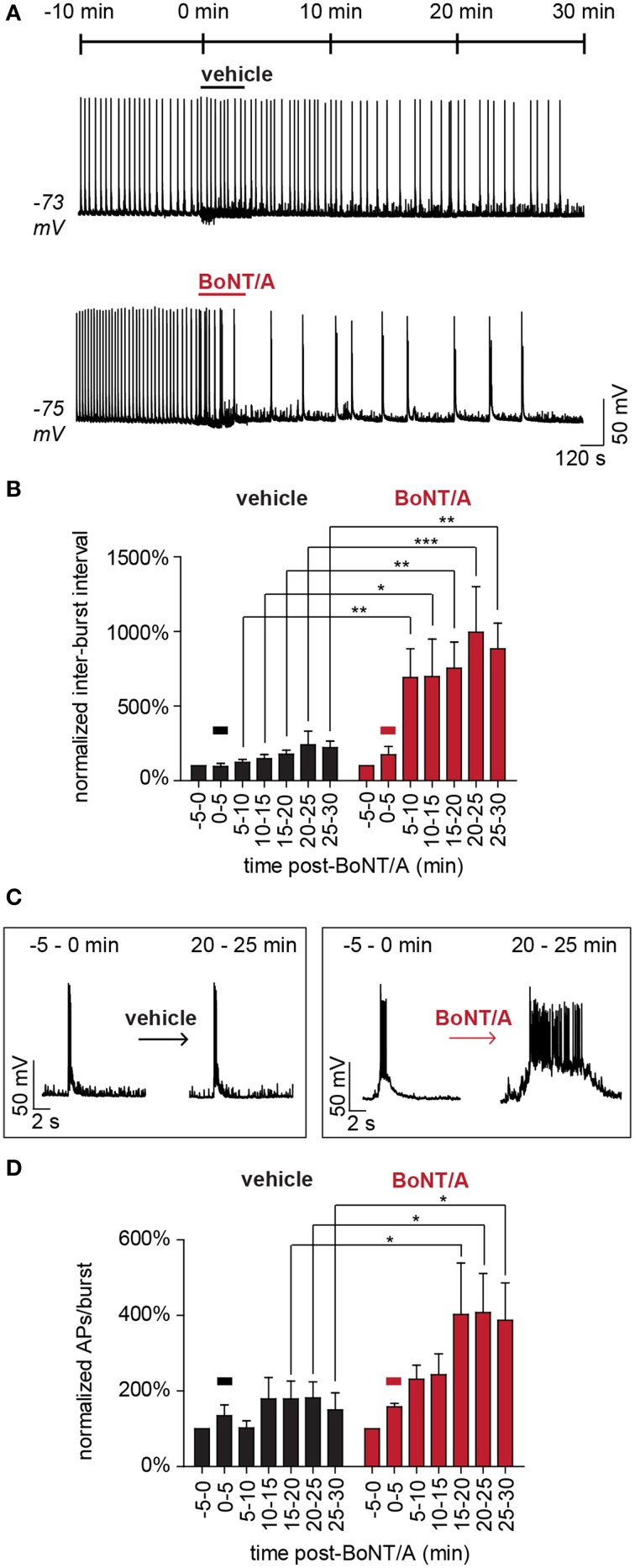
**Acute disinhibition of network activity following superfusion of BoNT/A. (A)** Representative current-clamp recordings demonstrating the acute effects of superfusion with vehicle (ERB; top) or BoNT/A (ERB with BoNT/A; bottom) on AP bursting behavior (*n* = 7 each). **(B)** Comparisons of mean inter-burst intervals in vehicle-treated (black) and BoNT/A-treated (red) networks from −5 to 30 min after superfusion (*n* = 7 each). **(C)** Expanded time-scale illustrating representative bursting patterns before and after superfusion. **(D)** Comparisons of mean APs per burst in vehicle-treated (black) and BoNT/A-treated (red) networks from −5 to 30 min after superfusion (*n* = 7 each). In **(A,B,D**), the bar signifies superfusion. All data are normalized to baseline values calculated over the 5 min window immediately prior to superfusion (−5 to 0 min) and compared between equivalent time windows between treatment groups. ^*^ indicates a *P* < 0.05; ^**^ indicates a *P* < 0.01; ^***^ indicates a *P* < 0.001.

Continuous current-clamp recordings were used to indirectly measure the effects of accelerated GABAergic impairment on network activity following superfusion with 20 pM BoNT/A. To increase the power and sensitivity of this approach, network activity was normalized to baseline values measured 5 min prior to BoNT/A perfusion and compared to time-matched data from control superfusions. Readouts of network activity included normalized changes in inter-burst intervals, burst durations and APs per burst. To test for potential confounds, three different control treatments were evaluated: vehicle, 200 pM formalin-inactivated toxoid or 20 pM heat-inactivated BoNT/A (Figure 5A). There were no apparent differences among the control treatments in inter-burst intervals (IBI; Figure 5B) or the number of APs per burst over 30 min (Figures 5C,D). All controls exhibited a non-significant trend toward fewer APs and more sub-threshold EPSPs over time, presumably due to intracellular ionic changes caused by dialysis. Perfusion with BoNT/A significantly increased IBI and APs per burst within 10 min, confirming network disinhibition (Figures 5A–D). By 30 min after toxin addition, baseline firing patterns had changed from frequent, short bursts with average durations of 0.7 ± 0.2 s to epileptiform-like bursts lasting 3.6 ± 0.3 s (*p* < 0.01). Collectively, these studies confirm that BoNT/A addition selectively impairs GABAergic signaling within minutes, leading to the rapid disinhibition of network activity.

## Discussion

Using detailed electrophysiological and molecular studies, we demonstrate that networked neuron cultures derived from embryonic stem cells undergo impaired synaptic transmission in response to BoNT/A intoxication. Near-total silencing of synaptic activity was observed within 3 h of toxin addition and was not attributable to cytotoxicity or disrupted intrinsic electrical properties. Measurements of synaptic activity revealed that GABAergic synapses are intoxicated at least 7-fold more rapidly than glutamatergic synapses. These findings were corroborated by ICC studies indicating that SNAP-25 initially accumulates in GABAergic terminals, as well as in network-level assays showing acute disinhibition of spontaneous firing following toxin addition. Not only are ESNs the first cell-based model to replicate the *in vivo* pathophysiologies responsible for clinical botulism, but the presence of synaptically driven network responses also allowed elucidation of synapse- and network-level responses to intoxication with multiple levels of resolution.

An intriguing finding of this study was the identification of synapse-specific latencies of intoxication. In networks containing excitatory and inhibitory inputs, subtle changes in the E/I ratio can have large effects on network behavior (McCormick and Contreras, 2001). In ESNs, the accelerated intoxication of GABAergic synapses acutely disinhibits network behavior, driving a transition from stochastic firing to prolonged bursts, followed ultimately by network silencing as glutamatergic synapses are intoxicated. Observations of synapse subtype-specific latencies are consistent with *in vitro* observations of disinhibited network responses following addition of BoNT/A to primary cortical or spinal cord neurons on planar microelectrode arrays (Scarlatos et al., 2008; Pancrazio et al., 2014) as well as *in vivo* reports of seizures following the intrahippocampal administration of BoNT/B (Broer et al., 2013). The rapid elimination of GABAergic signaling by BoNT/A treatment also suggests that functional assays directly (MIST) or indirectly (network responses) measuring the integrity of inhibitory neurotransmission could be leveraged as highly sensitive, specific and rapid methods to detect the presence of active BoNT.

While previous studies have attempted to elucidate the rates of various steps associated with BoNT/A uptake and activation *in vitro*, none have exploited the critical balance of spontaneous network behavior to provide a sensitive metric of toxin activity. The short latency of GABAergic intoxication suggested that BoNT/A is rapidly internalized and activated in GABAergic synapses in spontaneously active neuron cultures. Previous studies have shown that binding of BoNT/A to cognate pre-synaptic receptors can occur in solution within 1 min (Vazquez-Cintron et al., 2014), while inhibitory neurotransmission was reported to be decreased in spinal cord synaptic bouton preparations within 15 min of BoNT/A addition (Akaike et al., 2010). Internalization of small quantities of BoNT/A are sufficient to paralyze whole nerve terminals (Sudhof and Scheller, 2003), in part attributable to the pre-synaptic accumulation of LC/A and the LC/A *k*_cat_ = 60 s^−1^ (Chen and Barbieri, 2006). Thus, while the rates of GABAergic intoxication were unexpectedly fast, these findings are not inconsistent with published studies exploring kinetic and mechanistic aspects of BoNT intoxication.

Based on these and other studies, we can postulate several potential mechanisms to explain neuron subtype-specific latencies of intoxication. First, rates of synaptic vesicle endocytosis are activity-dependent, such that exposure of neurons to BoNT/A under depolarizing conditions accelerates toxin uptake and SNAP-25 cleavage (Simpson, 2004; Vazquez-Cintron et al., 2014). Inhibitory neurons have been reported to have increased firing rates compared to excitatory neurons (Wu et al., 2005; Clayton and Cousin, 2009; Roxin et al., 2011; Becchetti et al., 2012), and therefore may take up toxin more rapidly. Second, differential expression of BoNT/A co-receptors could increase selectivity for GABAergic synapses. In cortical neurons SV2A expression is predominantly associated with GABAergic synapses while SV2B is predominantly associated with glutamatergic synapses (Yeh et al., 2010). While both isoforms can act as BoNT receptors, SV2A has a higher affinity for BoNT/A and therefore may increase toxin internalization into GABAergic terminals (Dong et al., 2006; Blum et al., 2012). Third, structural differences between GABAergic and glutamatergic neurons, such as morphology (Benson et al., 1994) or pre-synaptic structure (Kuzirian and Paradis, 2011), may also contribute to differential rates of intoxication.

Though cleavage of SNAP-25 is the molecular mechanism underlying BoNT/A-mediated paralysis, the relationship between the spatiotemporal production of cleaved SNAP-25 (cSNAP-25) and decremented synaptic function has been difficult to establish. *In vivo* studies conducted in mice and frogs suggest that cleavage of less than 12% of total cellular SNAP-25 is sufficient to reduce acetylcholine release to below threshold in neuromuscular junctions (NMJs) (Raciborska et al., 1998; Meunier et al., 2003), whereas *in vitro* studies conducted in immature primary neuron cultures have suggested a linear inverse correlation between K^+^-induced bulk release of glutamate and SNAP-25 cleavage (Foran et al., 2003). In ESNs, cSNAP-25 was found to accumulate specifically in the pre-synaptic compartment at early timepoints, ostensibly as a result of the pre-synaptic entry of LC/A. Furthermore, cleavage of less than 3% of total cellular SNAP-25 was found to be sufficient to silence over 70% of synaptic activity. Since SNAP-25 is axolemmally distributed, a large fraction of SNAP-25 is not directly associated with pre-synaptic compartments (Tao-Cheng et al., 2000). The disproportionate sensitivity of synaptic activity to cSNAP-25 production therefore leads us to propose that the specific cleavage of the small subpopulation of SNAP-25 that is involved in neurotransmitter release is sufficient to block synaptic transmission. This hypothesis is consistent with accumulation of cSNAP-25 at SYN1^+^ puncta after BoNT/A addition. It would furthermore explain the increased sensitivity of MIST compared to immunoblots, in which cleavage of the small fraction of total SNAP-25 that is accessible to LC in the pre-synaptic compartment may be undetectable by immunoblot. At later time points, diffusion of intact SNAP-25 into the nerve terminal would result in the increased production of cSNAP-25, ultimately reaching quantities detectable by immunoblot.

Although the increased sensitivity of MIST assays (10–30 min) vs. molecular readouts (1 h by ICC or 2 h by immunoblot) may be attributable to the comparatively poor sensitivity of immunoblot-based assays, we cannot exclude the possibility that BoNT/A also impairs synaptic release via mechanisms independent of SNAP-25 cleavage. For example, the interaction between LC/A and SNAP-25 may sterically interfere with the formation of the ternary complex, thereby preventing synaptic vesicle fusion and neurotransmitter release independent of SNAP-25 cleavage. We anticipate that recently developed catalytically defective BoNT/A variants will offer improved tools to more directly address this possibility (Vazquez-Cintron et al., 2014).

It has been argued that SNAP-25 is not required for GABAergic neurotransmission, based on bulk release studies from synaptosomal preparations in the presence of BoNT/A (Frassoni et al., 2005). While this may be true for certain GABAergic neurons, neurotransmission has been found to be SNAP-25-dependent in multiple primary GABAergic populations. For example, transfection of intact SNAP-25 into hippocampal neurons harvested from homozygous SNAP-25 knockouts has been shown to rescue GABAergic neurotransmission (Tafoya et al., 2006, 2008; Delgado-Martinez et al., 2007). Furthermore, intoxication with BoNT/A2, which targets SNAP-25, silences GABAergic and glycinergic neurotransmission in rat spinal cord synaptic bouton preparations (Akaike et al., 2010). Consistent with these studies, we found that intoxication with BoNT/A and cleavage of SNAP-25 in GAD1^+^ pre-synaptic terminals is correlated with the loss of post-synaptic detection of monosynaptic inhibitory events, confirming that intact SNAP-25 is necessary for GABAergic neurotransmission in ESNs.

GABAergic signaling involves inhibition of both pre-synaptic release probabilities via metabotropic GABAR_B_ and post-synaptic detection via ionotropic GABAR_A_ (Wang et al., 2003; Chalifoux and Carter, 2011). Although we did not directly isolate and quantify GABAR_B_ function in intoxicated neurons, the reduction in mIPSCs following BoNT/A addition suggests that GABAR_B_-mediated effects on neurotransmission may also be reduced. Since GABAR_B_ has a long-lasting, depressive effect on pre-synaptic release, impairment of GABAR_B_ signaling is likely to have contributed to the observed increase in mPSC detection at 30–49 min.

The NMJ, ganglia, and autonomic synapses of the PNS are considered the clinically relevant targets of BoNT intoxication. However, the possibility that peripherally administered BoNT can lead to central nervous system intoxication is currently being debated. Recent findings *in vitro* and *in vivo* suggest that BoNT/A may undergo retrograde transport and be released from motor neurons to intoxicate central synapses (Restani et al., 2012a,b; Marchand-Pauvert et al., 2013). Furthermore, the direct administration of BoNT has been proposed as a potential therapeutic for several indications, including epilepsy-induced seizures (Kato et al., 2013). The observation of subtype-specific latencies of intoxication in this study raises the possibility that intoxication of central neuronal networks containing excitatory and inhibitory synapses will result in complex, multiphasic behaviors in a dose- and time-specific fashion. The effects of CNS exposure to BoNT are likely to be dynamic and difficult to predict, influenced by factors such as spatial and temporal differences in toxin concentration, the functions of exposed neurons and local circuit topology. These findings also raise the possibility that altering dosing levels in a controlled fashion in specific brain regions may allow the purposeful modulation of particular neuronal circuits.

In conclusion, we have found that synaptically coupled, networked populations of ESNs undergo blockade of synaptic neurotransmission following exposure to BoNT/A, confirming that ESNs replicate the pathophysiologies that are responsible for the clinical manifestations of botulism. These studies validate the use of synaptically active and networked ESNs as a replacement for primary neurons in BoNT studies, offering a novel, biologically relevant, cost-effective, and scalable platform for the specific and sensitive study of toxin that does not require animal use or rely on non-neuronal models with dubious relevance. They furthermore indicate that synaptically coupled populations of stem cell-derived neurons offer a relevant model for therapeutic discovery, in which the functional effects of toxin:host interactions are highly conserved and thus likely to be translatable to the PNS. The application of synaptic function-based assays to functionally detect the presence of BoNT/A within minutes or hours of exposure is a significant improvement over existing techniques to measure active toxin *in situ*, which are slower and either less specific (e.g., the mouse lethality assay) or less sensitive (e.g., immunoblots). Finally, the specificity rendered by electrophysiological measurements of synaptic activity indicates that ESNs may be suitable for the study of other pre-synaptically targeted toxins, such as tetanus or the remaining BoNT serotypes. Exploitation of this scalable model for BoNT research will lead to better understanding of synaptic and network responses to intoxication and provide a relevant platform for rapid toxin detection, improved potency determination and development of medical countermeasures.

## Author contributions

PB and PM conceived and designed the experiments. PB and SS performed the experiments. PB, SS, MA, and PM analyzed and interpreted the data. PB, MA, and PM wrote the paper. PB, SS, MA, and PM granted final approval of the version to be published and agree to be accountable for all aspects of the work.

### Conflict of interest statement

The authors declare that the research was conducted in the absence of any commercial or financial relationships that could be construed as a potential conflict of interest.

## Abbreviations

AMPAR: α-amino-3-hydroxy-5-methyl-4-isoxazolepropionic acid receptor
AP: action potential
BoNT/A: botulinum neurotoxin serotype A1
BoNT: botulinum neurotoxin
CNS: central nervous system
cSNAP-25: cleaved synaptosomal-associated protein 25
DAP: days *in vitro*
E/I: excitatory/inhibitory
EPSP: excitatory post-synaptic potential
ESN: mouse embryonic stem cell-derived neurons
GABAR_A_: γ-aminobutyric acid receptor A
GABAR_B_: γ-aminobutyric acid receptor B
HC: heavy chain
IBI: inter-burst interval
LC: light chain
LC/A: light chain of botulinum serotype A
mEPSC: miniature excitatory post-synaptic current
MLA: mouse lethality assay
mIPSC: miniature inhibitory post-synaptic current
MIST: measured inhibition of synaptic transmission
NMDAR: *N*-Methyl-D-aspartate receptor
NMJ: neuromuscular junction
PNS: peripheral nervous system
T50_i_: time to 50% inhibition
TTX: tetrodotoxin.
