# Supplementary figures and images for: Accelerated intoxication of GABAergic synapses by botulinum neurotoxin A disinhibits stem cell-derived neuron networks prior to network silencing
Source: Front Cell Neurosci. 2015 Apr 23;9:159. doi: 10.3389/fncel.2015.00159 (PMC4407583; doi:10.3389/fncel.2015.00159)

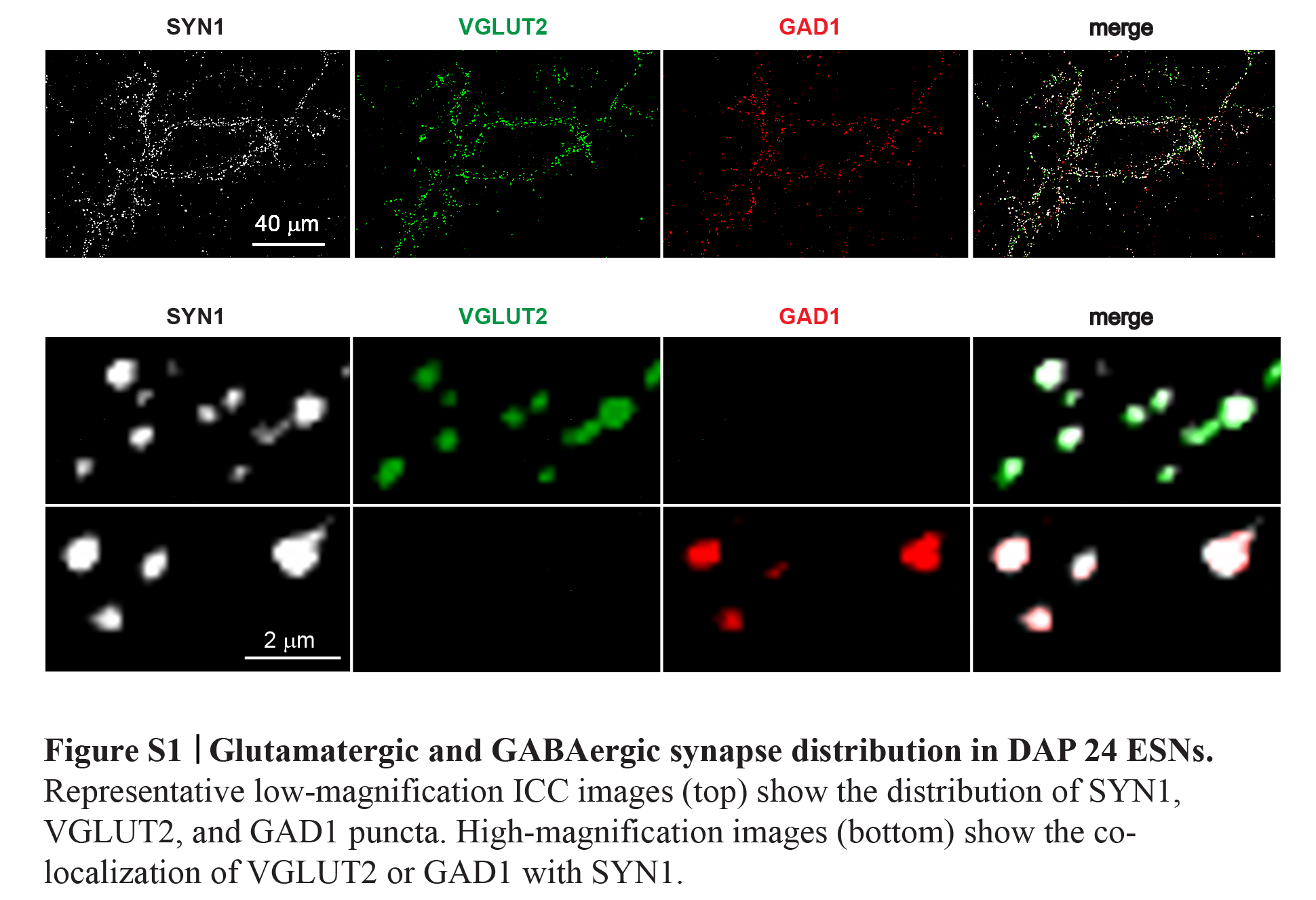

Supplement: Supplementary file 1 [file Image1.TIF]

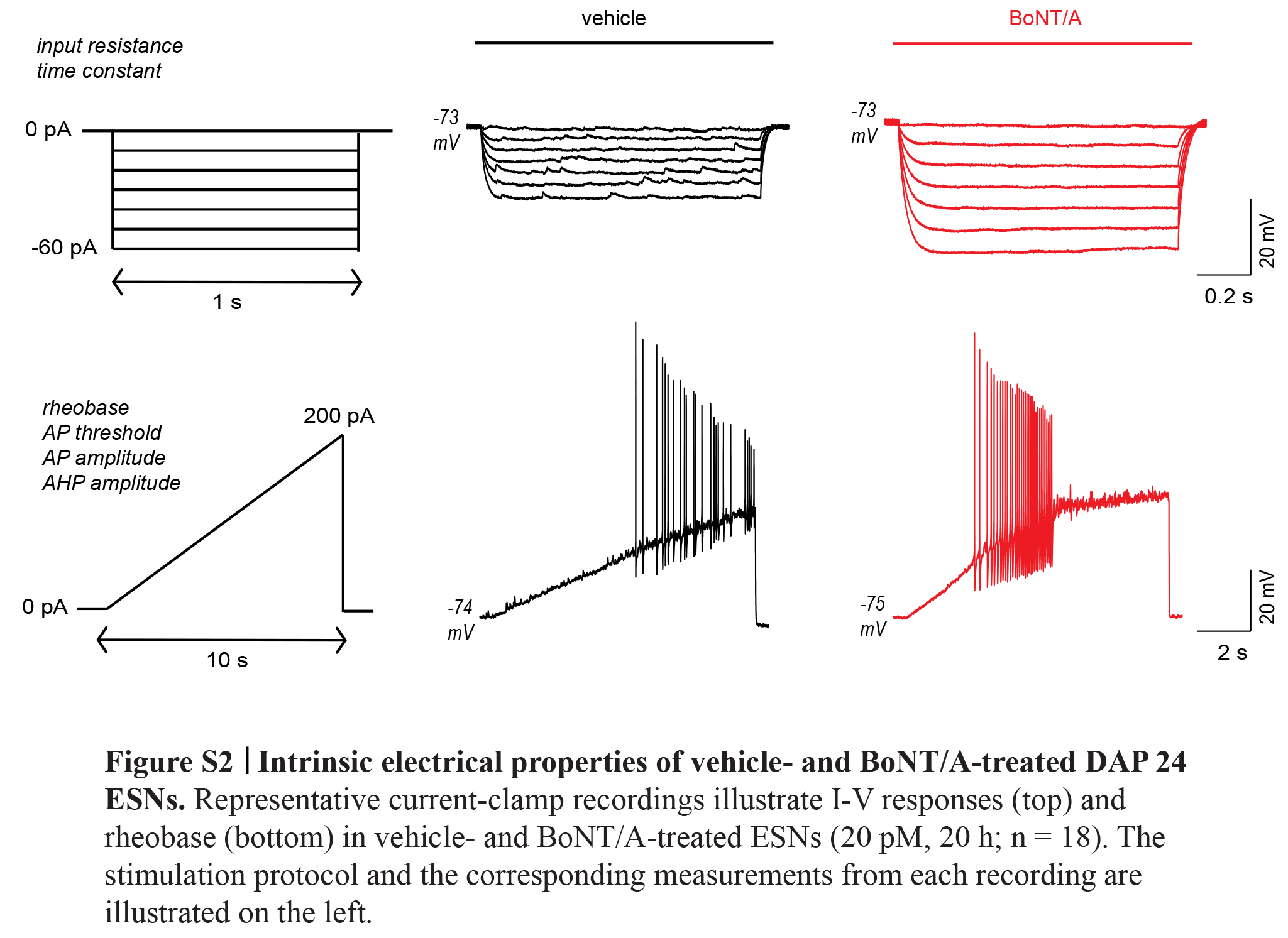

Supplement: Supplementary file 2 [file Image2.TIF]
